# Shame in sport: tracing the past and looking to the future

**DOI:** 10.3389/fpubh.2026.1816837

**Published:** 2026-06-09

**Authors:** Hui Huang, Xiangbo Wang, Houjin Chen, Jie Chen

**Affiliations:** 1 College of Physical Education and Health, Guangxi Normal University, Guilin, China; 2 College of Sports, Hunan Agricultural University, Changsha, China; 3 College of Physical Education, Woosuk University, Jeonju, Republic of Korea; 4 School of Sports Science, Changsha Normal University, Changsha, China

**Keywords:** antecedents, body shame, consequences, outcomes, sports shame

## Abstract

Shame serves as a crucial perspective for investigating individual sports withdrawal. Over the past two decades, this research topic has boomed rapidly in the field of social psychology of sport, with scholars conducting systematic explorations on its measurement tools, antecedent variables and action mechanisms. However, existing studies lack a systematic sorting and comprehensive understanding of its process mechanisms. First, this paper combs the research evolution of shame in sport, clarifies its evolutionary trends, and summarizes the limitations of existing measurement scales. Second, it generalizes the influencing factors of shame in sport from the perspectives of behavioral subjects and behavioral environments. Third, it clarifies the double-edged sword effect of shame on individuals in the field of sport and its contingency moderating factors. On this basis, the paper finally discusses the key problems and challenges faced by this research field, and focuses on the future research directions, aiming to theoretically deepen and expand the research content of shame in sport, and provide evidence-based references for improving sports participation and mental health in practice.

## 1 Introduction

Sport is inherently a highly visible social context where participants are constantly observed, evaluated and compared by others. As such, sports not only offer numerous opportunities for individuals to demonstrate their personal abilities and achievements, but also harbor the potential for shame experiences triggered by failure, mistakes or norm violations (1). For instance, shame may emerge when a student makes a mistake while being asked by a teacher to perform publicly in a physical education class, is mocked for their physique or sports attire not conforming to mainstream esthetic standards, or finds their progress slow in learning a new sports skill while others around them perform excellently. It should be noted that among self-conscious emotions, shame and guilt are two concepts often confused with each other. Though they overlap in emotional experience, they are essentially distinct emotions. Shame arises when individuals perceive flaws in their own competence, character or self-worth, accompanied by a strong tendency of self-denial. By contrast, guilt focuses more on behavioral consequences, referring to feelings triggered by one’s own actions, and is usually accompanied by the desire to make amends to ease inner remorse (2). Besides, embarrassment is also frequently mixed up with shame. Yet in most daily sports scenarios, embarrassment is a socially induced emotional state that is highly situation-dependent and transient, while shame exerts more lasting psychological harm (3). Given that sports serve to publicly display personal abilities and achievements rather than judge daily moral conduct, shame is more prevalent and typical than guilt in athletic contexts. Since embarrassment has far less profound long-term impacts on individuals’ mental health than shame does, this study takes shame as its core research subject. In this research, the term shame refers to a complex negative emotional experience that arises in an individual within a sports context. It is triggered by discrepancies between one’s perceived physical ability, athletic competence, or performance and social or self-expectations, or induced by others through words, expressions, postures, and other means. Such an experience encompasses feelings of distress, aversion, embarrassment, and a sense of personal inadequacy.

Over the past three decades, numerous researchers have gradually turned their attention to shame in sport, yet relevant studies remain relatively fragmented. This paper aims to synthesize and discuss these existing researches: first, it will elaborate on the research evolution of shame in sport and the iteration of its measurement tools; subsequently, it will shift to an overview of the influencing factors of shame; then, it will expound on the consequences and effects of shame; finally, it will identify the core problems and challenges facing this research area, and accordingly propose future research directions. It is noteworthy that although several fragmented reviews on body shame have emerged sporadically, but body shame is only a part of the research on shame in sport. Therefore, this paper does not intend to fill gaps based on existing reviews, but rather provides the first integrated overview of shame in sport. Furthermore, this paper is not an exhaustive comprehensive review covering all relevant studies; instead, it aims to present the overall landscape of research on shame in sport, while appropriately incorporating the latest theoretical and empirical findings, so as to offer directions and insights for subsequent studies.

## 2 Research evolution and measurement iteration

### 2.1 Research evolution of shame in sport

Shame in sport has been an academic research topic for more than three decades, during which both research and practical contexts have undergone constant iteration and transformation. To gain a more comprehensive understanding of the research on shame in sport, this paper divides such research into three stages based on the academic community’s insights into its developmental course, combined with the evolution of research methods and research themes (Table 1).

**Table 1:** Theoretical evolution of shame research in sport.

Stage	Representative	Core contributions	Main limitations
Theoretical Foundation Period (1978—2000)	Kohut (79)	Proposed the concept of “damaged self-structure”, laying a foundation for understanding the mechanism of in-depth self-deprecation	Failed to directly involve physical experience
	Mckinley and Hyde (4)	Put forward objectified body consciousness, pointing out that women internalize external esthetic standards as a source of self-monitoring	Focused on static physical appearance, mainly targeting women’s daily life
	Fredrickson and Roberts (5)	Defined self-objectification as an emotional structure	Failed to distinguish situational differences and ignored the functionality of the body in sport
Situational Transition Period (2001—2008)	Parsons and Betz (43)	First applied objectification theory to women’s sports participation, marketing the shift of body shame research from static perspective to sports perspective	Still centered on appearance-oriented sports, without involving sports performance
	Muscat & Long (6)	Indicated that the highly evaluate sports environment strengthens body monitoring and induces shame and anxiety	Restricted to women’s physical appearance, ignored identity recognition and social evaluation
Multi-dimensional Expansion Period (2009 to present)	Partridge & Elison (80)	First systematically defined shame emotions in sport contexts	Lacked conceptual operational procedures and measurement indicators
	Fontana and Fry (10)	First explicitly proposed the concept of sports shame, distinguishing outcome shame and process shame	As an emerging concept, it lacks sufficient empirical verification and cross-cultural applicability tests
	Pila et al. (7); Zhang et al. (8)	Further deepened the objectification perspective and explored the correlation between social comparison and body shame	Insufficient integration with other theoretical perspectives

First, the theoretical foundation stage. Research in this stage focused primarily on the psychological structure and occurrence mechanism of shame emotion, without examining it in the specific context of sports. In 1978, Kohut proposed the concept of self-structure impairment, which provided a unique perspective for understanding shame (79). Though this theory did not directly address the physical body, it laid a foundational groundwork for subsequent research on bodily shame. In the 1990s, feminist psychology began to link shame with bodily experiences. Based on objectification theory, McKinley and Hyde (4) developed the framework of Objectified Body Consciousness, arguing that women are perpetually positioned as the watched in social culture, thus internalizing external esthetic standards as an ideal body image and developing persistent body surveillance and bodily shame. Fredrickson and Roberts (5) further pointed out that such self-objectification is not merely a cognitive tendency but an emotional structure, leading women to experience shame when they fail to conform to social bodily norms. Research in this stage established the theoretical foundation for bodily shame, yet failed to distinguish between contextual differences. The second is the contextual migration stage. In the 21st century, research on bodily shame began to migrate from the context of daily life to that of sports participation. In 2001, Parsons and Betz (43) became the first scholar to apply objectification theory to the study of women’s sports participation, finding that while women gain psychological empowerment from sports, they may also experience heightened bodily shame due to appearance-oriented sports types. This study marked the shift of bodily shame research from the static body to the body in motion, and initiated investigations into how shame is activated and reproduced in sport contexts. Subsequently, Muscat and Long (6) further indicated that the high evaluative nature of sports environments reinforces women’s tendency toward body surveillance, leading them to perceive their bodies as objects to be watched and thus inducing shame and anxiety. While research in this stage expanded the contextual boundaries of bodily shame, it focused mainly on the correlation between women’s bodies and appearance evaluation, and failed to fully explore the multidimensional functions of shame in athletic performance, identity construction and social interaction. Thirdly, the multi-dimensional expansion stage. Over the past decade, research on shame in sport has entered a phase of parallel theoretical deepening and contextual refinement, showing a trend of multi-axial expansion. On the one hand, research has continued the objectification perspective to explore the relationships between physical appearance, social comparison and shame (7, 8). On the other hand, research has extended the study of shame from the physical body to athletic performance and identity construction, emphasizing that shame in sport contexts stems not only from the body as an object, but also more likely from an individual’s competence, social identity and sense of belonging (1, 9). In 2009, Partridge and Elison (80) systematically defined shame within the context of sport; though they did not explicitly propose the term sport shame, they laid the theoretical and empirical foundation for its subsequent conceptualization. Building on this, Fontana and Fry (10) became the first to explicitly put forward the concept of Shame in Sport, distinguishing it from general shame emotion and measuring and validating it as a specific self-conscious emotion in sport contexts. This categorization not only continued Partridge et al. (9) focus on the functionality of shame coping, but also extended such research to the emotion generation stage, emphasizing that the content and sources of shame themselves carry distinct social and psychological meanings. Therefore, research on shame in sport is still in a phase of rapid development, and current studies have initially established a framework for sport shame as a context-specific emotion.

### 2.2 Measurement iteration of shame in sport

Research on the measurement of shame in sport started relatively late. Although relevant scholars began to pay attention to shame experiences in the contexts of physical activity and self-awareness as early as the 1980s, the subsequent two decades saw research remain largely at the theoretical analysis level, lacking substantial empirical studies. With the continuous development of sports and psychology disciplines, scholars have begun to explore how to measure shame in sport. Corresponding to the theoretical development, the iteration of measurement tools for shame in sport has also gone through three stages (Table 2).

**Table 2:** Iteration of shame measurement tools in sport.

Stage	Year	Scale name (Abbreviation)	Developer	Measurement content	Main limitations
Criterion-referenced Tool Stage	1989	Test of Self-Conscious Affect (TOSCA)	Tangney et al.	Shame and guilt tendency in moral and social contexts	Items involve daily moral events irrelevant to sports scenarios, only applicable as calibration criteria
Body-situational Measurement Tool Stage	1996	Objectified Body Consciousness Scale (OBCS)	McKinley & Hyde	Objectified body consciousness and body shame	Focuses on social esthetic criteria, excluding physical function and athletic performance
	1998	Body Shame Questionnaire (BSQ)	Noll & Fredrickson	Infers body shame indirectly through willingness to alter body parts	Indirect measurement, non-sports setting, no direct assessment of emotional experience
	2002	Experience of Shame Scale (ESS)	Andrews & Qian	Behavioral, physical and character-related shame	Lacks sports specificity, fails to capture unique inducing factors in sport contexts
	2003	Body Image Guilt and Shame Scale (BIGSS)	Thompson et al	Shame and guilt related to body image	Distinguishes body shame and guilt systematically yet confined to static body image framework
	2007	Weight-and Body-Related Shame and Guilt (WEB-SG)	Conradt et al	Shame and guilt triggered by public physical observation	Concentrates on obesity and avoidance behaviors, insufficient reflection on sports participation
	2014	Body Image Shame Scale (BISS)	Duarte et al	Body image shame	Does not cover physical functional performance and social comparison in sport
	2016	Body-Focused Shame and Guilt Scale (BF-SGS)	Weingarden et al	Shame arising from gaze on specific body parts	Designed for body dysmorphic disorder populations with narrow regional specificity
The stage of sports-situational measurement tools	2017	Shame in Sport Questionnaire (SSQ)	Fontana & Fry	Outcome shame and process shame	Developed targeting athletes, cross-cultural applicability unvalidated
	2021	Athletic Perceptions and Performance Shame Scale (APPS)	Rice et al	Ability shame, identity shame and social comparison shame	Developed targeting athletes, cross-cultural applicability unvalidated

First, the criterion-referenced measurement stage. Early measurement tools for shame in sport were mostly general scales. A typical example is the Test of Self-Conscious Affect (TOSCA) developed by Tangney et al. (11), which measures individuals’ proneness to shame and guilt in moral or social contexts. Though the scale distinguishes between shame and guilt, its items are set in daily moral scenarios that are irrelevant to sports, thus serving merely as a criterion reference. Second, the stage of body-situational measurement tools. Based on feminist social construction theory, McKinley and Hyde (4) developed and validated the Objectified Body Consciousness Scale (OBCS) to assess the shame arising from individuals’ failure to meet social esthetic standards for the body, and proposed objectified body consciousness as the core mechanism for understanding women’s negative bodily experiences. To test objectification theory, Noll and Fredrickson (12) created the Body Shame Questionnaire (BSQ), which infers bodily shame indirectly by measuring participants’ desire to change various bodily parts/attributes and the intensity and frequency of such desires, shifting the study of shame from moral to bodily contexts. The Experience of Shame Scale (ESS) developed by Andrews and Qian (13) was one of the early tools attempting to distinguish the dimensions of shame, including behavioral shame, bodily shame and characterological shame, and it provided a structural reference for the development of subsequent shame scales in sport. Building on this, Thompson et al. (14) developed the Body Image Guilt and Shame Scale (BIGSS), which for the first time systematically distinguished and measured shame and guilt in body image, separating bodily shame and bodily guilt from general moral emotions. This enabled researchers and clinicians to accurately measure individuals’ shame and guilt experiences in the specific domain of the body, and provided a template for the subsequent adaptation of scales to sports contexts. Specialized scales for bodily contexts did not emerge until 2007, when Conradt et al. (81) developed the Weight-and Body-Related Shame and Guilt Scale (WEB-SG), which anchored its scenarios in publicly visible situations of bodily gaze and served as a specialized tool for research on obesity and sports avoidance behavior. Research became further refined in the 2010s. Duarte et al. (15) developed and validated the Body Image Shame Scale (BISS) to assess the shame individuals experience regarding their body image, and classified shame into external shame (devaluation by others) and internal shame (self-loathing). Weingarden et al. (16) compiled the Body-Focused Shame and Guilt Scale (BF-SGS) for individuals with body dysmorphic disorder (BDD); its items focus on the shame felt when specific bodily parts (e.g., nose, skin, and chin) are stared at by others, supplementing a dimension of body part specificity. Third, the stage of sports-situational measurement tools. With the deepening of research, measurement tools for bodily contexts could no longer fully meet scholars’ research needs on shame in sport, prompting relevant scholars to shift their focus from bodily contexts to identity and performance. For example, Fontana and Fry (10) compiled the Shame in Sport Questionnaire (SSQ), challenging the oversimplified logic that “shame = an emotion of failure”. They proposed that athletes may experience another type of shame with distinct functional implications—process shame—stemming from “not having tried one’s best”, thus distinguishing result shame from process shame at the conceptual level for the first time. Rice et al. (17) argued that the “moral or social scenarios” in general shame scales poorly align with the real-life scenarios of athletes and neglect high-frequency painful points such as “being outperformed by teammates” and “letting family expectations down”. They proposed dividing sport shame into three dimensions: competence shame, identity shame and comparative shame, and on this basis developed the Athletic Perceptions and Performance Shame Scale (APPS) to capture shame rooted in athletes’ identity. In conclusion, the measurement of shame in sport has completed an iteration from general social/moral shame to bodily (image) shame, and then to sport-specific shame. This evolution reflects the academic community’s shift in understanding shame experiences in sport contexts—from a universal psychological perspective to the exploration of sports-specific dimensions.

## 3 Influencing factors of shame in sport: a multi-perspective comprehensive impact

**Figure 1:**
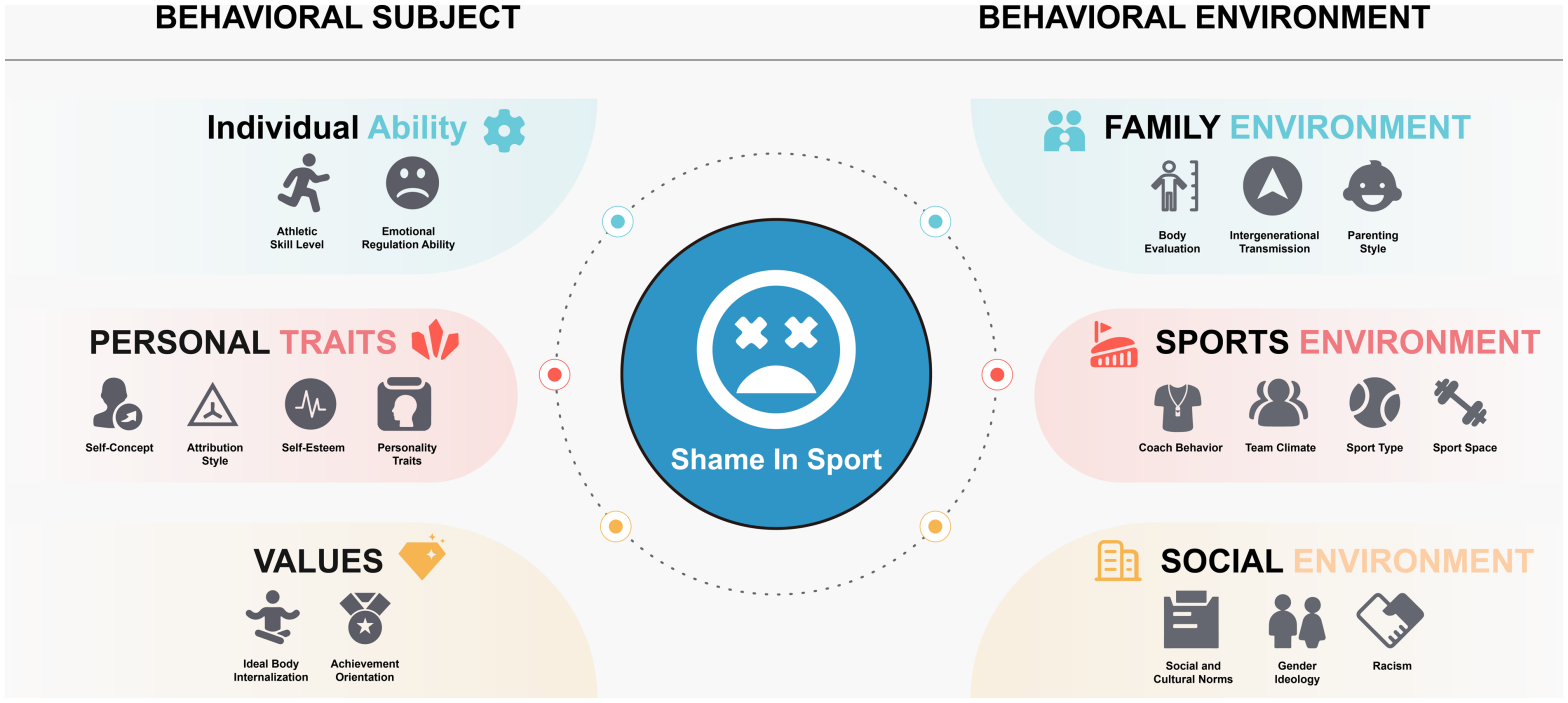
Multidimensional factors influencing shame in sport.

The influencing factors of shame in sport have attracted extensive academic attention, with existing explanations focusing primarily on the roles of individual internal psychological structures and external environments. By sorting out the existing research findings, this paper summarizes the influencing factors of shame in sport from the perspectives of behavioral subjects and behavioral environments (Figure 1).

### 3.1 Influencing factors of behavioral subjects

#### 3.1.1 Individual competence

Social cognitive theory posits that individuals’ perceived capability to perform a behavior and judgment of behavioral outcomes exert a profound impact on their conduct (18). Within the research framework of shame in sport, competence refers to individuals’ comprehensive qualities to perceive and actually possess the ability to complete sports tasks or meet physical demands in sport; competence level determines the likelihood of individuals exposing failures in public situations. Individuals with insufficient competence are more prone to setbacks or poor performance in sport, which further triggers self-deprecation and anxiety about social evaluation, thus forming shame experiences. Existing studies mainly reveal the influence of individual competence from the perspectives of sports skill level and emotion regulation ability. Sports skill level refers to the technical proficiency and performance quality demonstrated by individuals in completing specific sports tasks or movements. Sports identity is largely built on the basis of successful athletic performance, and individuals with failed athletic performance or insufficient sports competence are more likely to generate shame (19). For instance, in the study by Rice et al. (17), adolescent athletes’ shame did not stem from physical appearance but from the core self-negation of “I am not good enough as an athlete”, namely their sports skill level failing to meet self or others’ expectations. Emotion regulation ability refers to individuals’ capacity to manage emotional experiences and expressions. Individuals with strong emotion regulation ability can quickly identify and accept shame emotions when encountering sports failures. In the study by Burychka et al. (20), self-compassion, as a core strategy of emotion regulation, provides a gentle and effective path for “identifying-accepting-transforming” shame. When individuals encounter failures in sport and generate shame, those with high self-compassion will initiate mindful awareness, place negative events in the framework of common humanity, and replace self-aggression with self-kindness, thereby blocking the generalization of shame. The study by Wollast et al. (21) further confirms that individuals with high self-compassion levels experience less intense shame even when facing physical surveillance. The study by Lamont (22) also indicates that the higher the level of mindfulness, the lower the body shame, and the stronger the ability to perceive and accept physical signals. In other words, the key reason why individuals with strong emotion regulation ability can quickly identify and accept shame lies not in “suppressing” emotions, but in transforming shame into self-care from a non-judgmental perspective, thus returning to trust in self-body and focus on bodily functions more rapidly.

#### 3.1.2 Personal traits

Personality trait theory holds that traits are the fundamental characteristics determining personal behaviors, and individuals’ psychology and behaviors are influenced by stable traits (23). In sport shame research, individual trait factors such as self-concept, attributional style, self-consciousness, self-esteem and personality traits have been widely explored. First, in terms of self-concept and attributional style, Tracy and Robins (24) pointed out in the theoretical model of “Self-Conscious Emotions” that self-conscious emotions like shame are not the direct outcomes of events themselves, but the results of how individuals attribute events and interpret the relationship between events and the self, emphasizing individuals’ subjective interpretation and evaluation of external events in the emotional generation process. The study by Crocker et al. (25) provides empirical evidence for this: it reveals that physical self-concept and shame proneness partially predict women’s body shame through global and controllable attributions. Second, self-esteem is a pervasive motivational force within individuals. Iannaccone et al. (26) argued that low self-esteem may induce a sense of ineffectiveness in individuals; when this sense is directed at the body, it manifests as body shame, meaning low self-esteem intensifies individuals’ body shame. Finally, in the dimension of personality traits, shame is regarded as one of the core emotions of narcissistic personality (27). Boursier and Gioia (28) found that individuals with vulnerable narcissism tend to base their self-worth on appearance, thus experiencing body shame, while those with grandiose narcissism may cover up inner body shame through external showing-off and self-presentation. Further, Alcaraz-Ibáñez et al. (29) revealed that the Big Five personality traits significantly predict body shame: neuroticism positively predicts body shame, whereas extraversion, conscientiousness and openness negatively predict it. After controlling for gender, age and weight differences, personality accounts for up to 26% of the variance in body shame. This indicates that stable personality tendencies are important antecedent variables of shame experiences in sport contexts, and also provides new evidence for the applicability of trait theory in the field of self-conscious emotions.

#### 3.1.3 Values

Values represent individuals’ cognition, comprehension, judgment and choices, serving as a guide for individual behavioral motivation. As individuals’ deep-seated beliefs, values not only determine goal-setting and behavioral priorities in sport contexts, but also provide internalized value criteria for their shame experiences. Existing studies mainly explore its impacts from two dimensions: ideal body internalization and achievement orientation. Ideal body internalization refers to individuals’ internalization of ideal body standards or appearance-valuing values (30). According to objectification theory, when women perceive their bodies do not conform to internalized body ideals in sport, intense body shame will be triggered (5). Huang et al. (31) further confirmed that ideal beauty internalization and self-objectification play a crucial mediating role between online objectification experiences and body shame. In other words, the key to body shame lies not in the objective physical form, but in its consistency with individuals’ internal standards of body and appearance. Achievement orientation refers to individuals’ value orientation of pursuing achievements or success to prove their abilities to society. Bloodworth and McNamee (32) found that young athletes experience value conflicts between the moral ideal of “clean competition” and realistic pressures; once failing to meet ideal standards (e.g., doping use), they will generate intense moral shame. Similarly, the analysis of Laine (33) on Finnish doping scandals indicated that the values of national identity and sports purity led the whole society to produce collective shame toward scandal participants, reflecting the role of values in constructing shame at the collective level.

### 3.2 Environmental influencing factors of behavior

#### 3.2.1 Family environment

Ecological systems theory posits that the family is the most immediate microenvironment affecting individual development and plays a pivotal role in the process of individual socialization (34). Based on a review of existing studies, the family environment exerts an impact on individuals’ shame experiences in sport contexts mainly through physical evaluation, intergenerational transmission and parenting styles. First, regarding physical evaluation, multiple studies have shown that direct evaluations of individuals’ body image, weight or sports performance by family members constitute an important external source of body shame and sports avoidance. For instance, Domoff et al. (35) indicated that negative body talk between mothers and daughters significantly intensifies body shame among adolescent girls. Pecini et al. (36) found that parental focus on appearance contributes to higher levels of body shame in children aged 7 to 12. Second, both social learning theory (37) and the intergenerational transmission effect (38) point out that parental behavioral characteristics are transmitted to children in daily life. Czepczor-Bernat et al. (39) demonstrated that parents’ own body dissatisfaction and perfectionism tendencies are significant predictors of children’s body shame levels. Rivero et al. further extended the scope to the entire family system, revealing that negative dietary or weight-related information from fathers, mothers and even sisters can independently and significantly predict body image shame among Latinx female college students. Finally, parenting styles also profoundly influence individuals’ shame in sport contexts. Gouveia et al. (40) proposed that parental mindful parenting practices can indirectly reduce adolescents’ body shame by enhancing their self-compassion. Using a 12-month longitudinal study, Mendes et al. (41) further confirmed that fewer early warm memories correlate with greater fear of others’ compassion and faster increases in body image shame among girls, which conversely indicates that positive parenting styles can block the development of body shame. In summary, the family environment is a multi-dimensional influencing system that can profoundly affect individuals’ shame in sport domain through multiple mechanisms.

#### 3.2.2 Sports environment

Scholars have explored the impact of the sports environment on individuals’ shame domain from diverse perspectives and achieved fruitful results. Based on research contents, the influence of the sports environment on individuals’ shame is mainly reflected in the following aspects: First, teachers’ or coaches’ evaluation and feedback methods. The language, attitude and feedback methods of teachers or coaches directly affect individuals’ sports shame experiences. Oliveira (42) found that when athletes perceive coaches’ compassion—i.e., responding to their failures and vulnerabilities with understanding, acceptance and support—their shame and self-criticism decrease significantly. Second, team climate. Fontana et al. (10) clearly indicated that athletes’ perception of team motivational climate directly affects their shame types; the caring climate that emphasizes care can effectively reduce athletes’ outcome shame caused by competition failure. Conversely, the task-involving climate and ego-involving climate that emphasize winning, losing and competition will significantly intensify athletes’ outcome shame and process shame. Third, sports types. Parsons (43) distinguished the impacts of different sports types on shame, revealing that sports emphasizing functionality or physical competence may bring higher psychological empowerment, while sports emphasizing appearance or conformity to traditional feminine esthetics are more likely to intensify women’s body shame. Fourth, sports spaces. Sports spaces directly affect the degree of individuals’ physical exposure and the frequency of shame triggers. Lim (44) pointed out that being evaluated for competitive failure in public is a core source of athletes’ shame. For children and adolescents, physical education classes and sports competitions all constitute such “gaze fields.” Agnes (45) and Huellemann et al. (46) also indicated that places requiring physical exposure such as locker rooms and swimming pools are high-incidence areas of shame, especially for transgender people, early adolescents or those dissatisfied with their body shapes. In summary, the sports environment systematically shapes individuals’ sports shame through multiple pathways including feedback methods, team climate, sports types and sports spaces.

#### 3.2.3 Social environment

Affective social constructionism posits that social norms constrain the emergence and development of individual emotions (47). Existing studies suggest that sociocultural norms, gender ideologies, and racism collectively construct a bodily field of gaze, evaluation, comparison, and discipline, thereby inducing or exacerbating shame in sport contexts. First, sociocultural norms surrounding ideal body standards constitute a crucial source of shame for individuals in sport settings. Through an analysis of celebrity gossip magazines, Hirdman (48) argued that such magazines frame body shame as a familiar emotional experience closely tied to femininity by showcasing female celebrities’ physical imperfections; they universalize physical failure by exposing flaws, thus generating widespread body shame and anxiety among readers. Empirical research by Manago et al. (49) found a significant positive correlation between Facebook use and body shame, as constant comparison between one’s own body and others’ curated images intensifies personal body dissatisfaction. Muscat and Long (6) revealed that critical weight-related comments in the social environment trigger stronger shame among female athletes and sports participants. Huellemann et al. (46) pointed out that female athletes often face tension between the “athletic body” and the “societal thin ideal” during identity transitions such as off-seasons, retirement, and injuries. Second, traditional gender ideologies profoundly shape bodily experiences and shame perceptions of individuals across genders in sport contexts. The analysis of Pavlidis (50) on Australian media showed that female athletes’ failures are often exploited to reinforce their identity as “women” rather than “athletes,” with their bodies continuously scrutinized through the lens of gender stereotypes. The study of Agnes (45) on transgender individuals found that sports spaces are highly gendered, with their infrastructure and culture presupposing a binary male–female bodily framework; this renders transgender bodies non-normative and highly susceptible to triggering body shame. Finally, racist esthetic standards exacerbate bodily discipline and shame risks for ethnic minorities. Keum et al. (51) found in their study of Asian American men that “gendered racism” leads to significant body shame through the internalization of Western muscular ideals. The research of Cheng (52) on Asian American female college students similarly demonstrated that “internalized racism” is a significant predictor of body shame. Moreover, studies have revealed that strong ethnic identity does not function as a protective factor; instead, it amplifies the negative impact of appearance prejudice on body shame, indicating that under the dominance of mainstream culture, efforts to uphold ethnic identity may deepen individuals’ internal bodily conflicts and shame experiences.

## 4 The dual-edged sword effect: consequences of shame in sport

Existing studies have revealed divergent views among scholars on the functions of shame in the sport domain. Some argue that shame serves as a vital mechanism for individuals’ sports morality management and sports behavior adjustment, while others hold that sports shame (body shame) is significantly correlated with various psychopathological characteristics. This divergence reveals the complexity of shame’s functions in sport, making it particularly crucial to comprehensively understand the dual-edged sword effect of shame in this field.

### 4.1 Positive effects: the adaptive functions of shame

Existing studies have mainly explored the positive roles of shame in the sport domain from the perspectives of behavioral regulation and moral normativity. First, at the behavioral regulation level, previous research has shown that self-conscious emotions elicit two broad response patterns to personal wrongdoing: one is to actively face and repair the harm caused, and the other is to avoid the problem and attempt to protect or maintain one’s self-image (53). This indicates that shame is not the endpoint of negative emotional expression; instead, it may motivate individuals to reconstruct their self-identity. This view aligns with the emotional theory from Probyn (54), which posits that pride and shame are mirror emotions—shame can be transformed into pride and serve as a driving force against stigma. Czepczor-Bernat (39) provides empirical support for this: the study found that women with high body shame are more likely to engage in appearance-oriented sports. Similarly, Pila (7) revealed that anticipated shame actually increases physical activity, albeit with the motivation rooted in appearance management rather than health promotion. Second, at the moral normativity level, scholars such as McNamee (32), Taylor and Johnson (55) argue that shame can internalize external sports rules into individual behavioral norms. They contend that shame effectively deters athletes from using performance-enhancing drugs, upholds the spirit of sportsmanship, and prevents cheating and other improper behaviors. Meanwhile, when individual shame escalates into a collective emotion, its social normative power becomes even stronger. For instance, the analysis of Laine (33) on Finnish doping scandals suggests that in the aftermath of sports scandals, collective shame prompts profound societal reflection, thereby reshaping national identity and moral consensus.

### 4.2 Negative effects: the destructive impacts of shame

Shame is one of the self-conscious emotions exerting the most significant negative effects on individual self-regulation (56, 57). Compared with other self-conscious emotions, shame is more focused on the trait level of “the self” and is often closely associated with individuals’ attention to their own flaws (58). In sport contexts, shame exerts notable adverse effects on individuals’ sports motivation, behavioral health management and mental health (59). First, at the sports motivation level, when experiencing intense body shame, appearance shame or competence shame, individuals tend to adopt avoidance strategies to protect themselves, such as staying away from sports or engaging in passive sports participation. For instance, in the study by Pila et al. (7), frequent body monitoring among adolescent girls reduces physical activity through experienced shame. Hofseth et al. (60) also pointed out that athletes with higher shame proneness are more likely to employ self-handicapping strategies for self-protection, thereby indirectly hindering the development of sports skills. Second, at the behavioral health management level, body shame is a crucial factor inducing unhealthy behaviors such as eating disorders and dietary disturbances. It makes individuals ignore bodily signals and downplay health priorities, thus increasing the likelihood of making poor decisions that sacrifice health for appearance (22, 61). This view is supported by abundant empirical studies and is prevalent among female athletes, college students, adolescents and even overweight or obese individuals. For example, Muscat and Long (6) indicated in their study that intensified body shame directly leads to disordered eating behaviors in female athletes; Mensinger et al. (62) revealed that body shame not only acts as a psychological bridge linking weight stigma and eating disorders, but also causes individuals to avoid necessary healthcare by reducing bodily reactivity and health evaluation, forming a vicious cycle of health deterioration. Finally, at the mental health level, sports- or body-related shame is a significant predictor of psychological disorders such as depression and anxiety symptoms (13, 41, 63–66). This mechanism is particularly prominent in high-pressure environments; for example, in the study by Lima et al. (44), athletes exposed to long-term public scrutiny and evaluation develop high-intensity sports shame, which is more likely to trigger mental health issues including anxiety, depression and sports stress. Meanwhile, persistent sports shame experiences not only impair individuals’ sense of self-worth, but may also gradually generalize into overall self-negation, further inducing extreme consequences such as self-harm behaviors and suicidal ideation (67, 68). In addition, sports- or body-related shame may be associated with cognitive performance. Correlational and experimental evidence suggests that prolonged body focus enhances body-related shame and consumes individuals’ attentional resources, leading to poor performance in cognitive and motor tasks (5, 69–72), yet this claim has not been further verified.

### 4.3 Contingency of the dual-edged sword effect of shame

A dialectical understanding of the positive functions and negative impacts of shame in sport is of great significance for revealing their inherent essence. Based on a review of existing literature, studies have mainly explored the contingency of this dual-edged sword effect from two aspects: internal behavioral subjects and external environments. From the perspective of internal behavioral subjects, the possession of strong psychological traits by individuals serves as the boundary for the transformation of shame in sport contexts. When individuals have high emotional regulation capabilities (such as mindfulness, body compassion, and self-compassion), they can effectively identify and accept shame, avoiding being trapped in rumination over past events (73–75). Second, the motivation of behavioral subjects is also a factor influencing the effect of shame. When individuals’ sports motivation is function-oriented, or their attention to their own bodies and appearance is based on future expectations rather than present ones, they may transform shame experiences into sports motivation and actively participate in physical activities (7, 8). From the perspective of external environments, the provision of supportive feedback and a positive sports climate constitutes the main condition affecting the effect of shame. Regarding the sports climate, when individuals perceive themselves in a task-involving or ego-involving climate, they tend to emphasize effort and preparation more; thus, when they fail to achieve expected goals, they are more likely to experience process shame. In contrast, when individuals perceive a caring climate, they can face failure more bravely without being defined by it (10). Furthermore, for the broader social environment, body-inclusive cultures (such as anti-fat stigma cultures) and media-promoted ideal body standards profoundly shape individuals’ self-perception and evaluation in sport contexts, collectively determining whether shame is amplified or mitigated by inclusive cultures (76, 77).

## 5 Research prospects

Over the past three decades, research on shame in sport has achieved remarkable progress, going through the stages of theoretical exploration, contextual transfer and multi-dimensional development. Relevant findings have provided diverse perspectives for understanding shame in sport, yet with the evolution of the times, these results can no longer fully meet the demands of different sports contexts, nor objectively reveal the inherent essence of shame. This chapter will discuss the key issues and challenges faced by this field, and focus on elaborating the future research directions.

Most existing studies are based on social cognitive theory, personality trait theory, ecological systems theory and other frameworks, predicting shame in sport from the perspectives of individual factors, family environment, sports environment and social environment, while only a few have focused on the individual-situation interaction. However, as an emotion associated with situational triggers, the intensity, duration and behavioral consequences of shame are often determined by the real-time interaction between individual traits and specific situations. Future research needs to introduce theoretical frameworks with a stronger interactional perspective, incorporating individual differences and specific situations into predictive models simultaneously.

The antecedents and consequences of shame in sport do not maintain full cross-cultural consistency, which has drawn insufficient attention in existing research. From the perspective of cross-cultural psychology, Western individualistic cultures regard guilt as the predominant moral emotion and lay stress on individuals’ specific behaviors. By contrast, East Asian collectivistic cultures attach greater importance to the function of shame in maintaining social norms (78). For instance, when making mistakes in sport events, Western athletes tend to feel guilty by attributing failures to personal efforts or flawed strategic decisions. In comparison, Eastern athletes are more likely to experience shame, viewing errors as deficiencies in personal competence or behaviors that harm collective honor. Current studies and measurement scales concerning sports shame are predominantly established on Western cultural backgrounds and research samples. This brings about cultural deviation in conceptual applicability, making it hard to interpret the unique emotional experiences of individuals in collectivist cultures. Accordingly, future research urgently needs to conduct cross-cultural comparisons to explore similarities and differences in sports-related shame experiences between individuals from collectivist and individualist cultural backgrounds.

When shame manifests in sport contexts should be the core concern of current research on sports withdrawal. Existing studies have mainly identified training scenarios as the situational triggers of shame in sport, leading to the prevailing theoretical view that shame-inducing sports contexts are confined to training settings. However, any public sports situation may expose individuals to body gaze or devaluation of athletic (physical) competence, yet few studies have systematically documented or explored the trajectory of shame in these non-training contexts. Thus, future research should expand the scope of shame-triggering contexts from training scenarios to the full spectrum of sports-related life situations.

Research on shame in sport and the development of its measurement tools stem from the fact that general shame scales fail to capture shame in physical activity or sports contexts, leading to insufficient contextual sensitivity and ambiguous boundaries of the shame construct. To fully understand shame arising from physical factors and occurring during sports, relevant scholars have developed various measurement tools targeting shame experiences in physical activity and self-consciousness contexts, and administered them across different scenarios. Through the application of these tools, scholars have identified multiple factors potentially influencing individuals’ shame in sport and gained a relatively clear understanding of the potential consequences brought by such shame. Despite the considerable number of measurement tools, mismatches or incompatibilities between contexts and tools remain prevalent. Future research should adhere to an integrated research paradigm, conduct more scientific and specific conceptual definition and connotative interpretation of shame in sport, and develop more precise scales to more comprehensively capture the diverse manifestations of shame in sport.

To date, most studies on shame in sport have adopted a cross-sectional design, while longitudinal follow-up studies and experimental research are relatively scarce, with weaknesses in qualitative research design—only a few scholars have attempted to conduct qualitative analyses (1). Cross-sectional designs hold high value in the early stages of field exploration, but they limit causal inference between variables, meaning the relationships among variables remain unclear or ambiguous. Meanwhile, compared to the existing systematic intervention programs for other psychological constructs, research on shame in sport is still in the descriptive stage. A very small number of studies have attempted experimental interventions, but they failed to distinguish contextual specificity, making it difficult to provide effective and targeted recommendations. In the future, mixed-methods research designs can be adopted for comprehensive analysis, and longitudinal follow-up studies and experimental research can be conducted to clarify the causal chain of shame in sport. In addition, shame exhibits temporal characteristics of rapid attenuation and repeated retrospection, while traditional research designs struggle to capture the intra-day fluctuations and temporal cycles of individuals’ sports-related shame. Future studies can attempt to use diary methods or situational recall approaches to explore shame in sport.

Currently, research on shame has mainly focused on athletes and college students, yet its scope needs to be extended to individuals at other developmental stages. As an emotional reflection of individual self-consciousness, shame varies with the distinct self-consciousness characteristics across different age groups. For example, in childhood, individuals tend to fail to stably distinguish between “the self” and “the self in others’ eyes”, and shame during this period mostly stems from direct parental negation. Thus, future research should appropriately expand the research subjects to explore whether shame in sport exhibit stage-specific features or group-specific patterns.

## 6 Conclusion

This comprehensive review presents an overall overview of shame research in sport. The study holds that over the past three decades and more, research on shame in sport has achieved considerable progress. The theoretical evolution of the field has advanced from “emotional structure” to “situational mechanisms”, and from “physical appearance” to “sports performance and identity”. Measurement tools have evolved from general shame scales to specialized scales tailored to multiple contexts and populations, while research designs have gradually expanded to include longitudinal follow-up studies. Nevertheless, based on the above review, current research still has notable limitations in theoretical integration, methodological design and practical application. Theoretically, relevant studies have long been grounded in objectification theory and overly centered on female perspectives, failing to fully cover male and non-binary groups. In terms of research content, most studies focus on body shame, appearance shame and beauty shame, while paying insufficient attention to shame related to athletic competence, sports performance and identity recognition. Furthermore, prevailing theories and measurement scales are predominantly developed against Western individualistic cultural backgrounds, which restricts their applicability to shame research within collectivist cultures. Methodologically, cross-sectional designs dominate existing studies, with only a small number adopting longitudinal tracking and experimental intervention. This makes it difficult to capture daily fluctuations of individual shame and clarify relevant causal mechanisms. In addition, given that the narrative logic of current measurement tools derives largely from Western individualistic values, future research can explore indigenous connotations of shame in diverse regions to deepen academic exploration. Practically, few studies explore experimental interventions targeting shameful behaviors in sport. The limited relevant researches mainly focus on body image issues, ignoring shame triggered by athletic ability and personal identity. Meanwhile, research subjects are largely confined to athletes and college students, whereas children and adolescents are neglected. Accordingly, the developmental trajectory of shame among young people remains poorly understood. To sum up, these findings can serve as references for designing and verifying intervention programs aimed at boosting sports participation and reducing maladaptive behaviors such as withdrawal and avoidance in sport settings, but numerous questions remain unanswered and much content awaits further exploration. Only by breaking down the current boundaries of context, developmental stage, and methodology in existing research, and situating shame within individuals’ lived sports experiences, can we gain a deeper understanding of shame among different individuals in diverse sports contexts.
